# 3,3′-Dimeth­oxy-2,2′-[(4,5-dimethyl-*o*-phenyl­ene)bis­(nitrilo­methanylyl­idene)]diphenol

**DOI:** 10.1107/S160053681100506X

**Published:** 2011-02-16

**Authors:** Atefeh Sahraei, Hadi Kargar, Reza Kia, Islam Ullah Khan

**Affiliations:** a Chemistry Department, Payame Noor University, Tehran 19395-4697, I. R. of Iran; b X-ray Crystallography Lab., Plasma Physics Research Center, Science and Research Branch, Islamic Azad University, Tehran, Iran; c Materials Chemistry Laboratory, Department of Chemistry, GC University, Lahore 54000, Pakistan

## Abstract

The asymmetric unit of the title compound, C_24_H_24_N_2_O_4_, comprises two crystallographically independent mol­ecules *A* and *B*. The dihedral angles between the central dimethyl-substituted benzene ring and the two outer benzene rings are 49.5 (1) and 5.06 (11)° in mol­ecule *A*, and 42.55 (8) and 5.77 (9)° in mol­ecule *B*. In each mol­ecule, two strong intra­molecular O—H⋯N hydrogen bonds generate two *S*(6) ring motifs. The crystal structure is further stabilized by inter­molecular π–π [centroid–centroid distances of 3.591 (1)–3.876 (1) Å] inter­actions.

## Related literature

For standard bond lengths, see: Allen *et al.* (1987 ▶). For hydrogen-bond motifs, see: Bernstein *et al.* (1995 ▶). For the structures of some tetra­dentate Schiff base ligands, see: Kargar *et al.* (2009 ▶, 2010*a* ▶,*b* ▶); Kia *et al.* (2010 ▶, 2011 ▶).
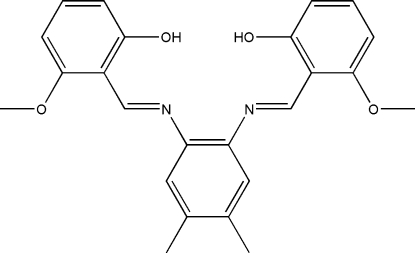


## Experimental

### 

#### Crystal data

C_24_H_24_N_2_O_4_
*M* *_r_* = 404.45
Triclinic, 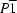

*a* = 8.0311 (2) Å
*b* = 12.5836 (3) Å
*c* = 20.6174 (5) Å
α = 86.900 (1)°
β = 82.549 (1)°
γ = 81.806 (1)°
*V* = 2043.57 (9) Å^3^
*Z* = 4
Mo *K*α radiation
μ = 0.09 mm^−1^
*T* = 296 K
0.32 × 0.15 × 0.11 mm

#### Data collection

Bruker SMART APEXII CCD area-detector diffractometer
Absorption correction: multi-scan (*SADABS*; Bruker, 2005 ▶) *T*_min_ = 0.972, *T*_max_ = 0.990
36786 measured reflections
10103 independent reflections
5848 reflections with *I* > 2*I*)
*R*_int_ = 0.044

#### Refinement

*R*[*F* ^2^ > 2σ(*F* ^2^)] = 0.053
*wR*(*F* ^2^) = 0.149
*S* = 1.01
10103 reflections
553 parameters
H-atom parameters constrained
Δρ_max_ = 0.30 e Å^−3^
Δρ_min_ = −0.20 e Å^−3^

### 

Data collection: *APEX2* (Bruker, 2005 ▶); cell refinement: *SAINT* (Bruker, 2005 ▶); data reduction: *SAINT*; program(s) used to solve structure: *SHELXTL* (Sheldrick, 2008 ▶); program(s) used to refine structure: *SHELXTL*; molecular graphics: *SHELXTL*; software used to prepare material for publication: *SHELXTL* and *PLATON* (Spek, 2009 ▶).

## Supplementary Material

Crystal structure: contains datablocks global, I. DOI: 10.1107/S160053681100506X/jh2267sup1.cif

Structure factors: contains datablocks I. DOI: 10.1107/S160053681100506X/jh2267Isup2.hkl

Additional supplementary materials:  crystallographic information; 3D view; checkCIF report

## Figures and Tables

**Table 1:** Hydrogen-bond geometry (Å, °)

*D*—H⋯*A*	*D*—H	H⋯*A*	*D*⋯*A*	*D*—H⋯*A*
O1*A*—H1⋯N1*A*	0.82	1.88	2.608 (2)	147
O2*A*—H2⋯N2*A*	0.82	1.81	2.541 (2)	148
O5*B*—H5⋯N3*B*	0.82	1.79	2.529 (2)	149
O6*B*—H6⋯N4*B*	0.82	1.89	2.621 (2)	148

**Fig. 1.**
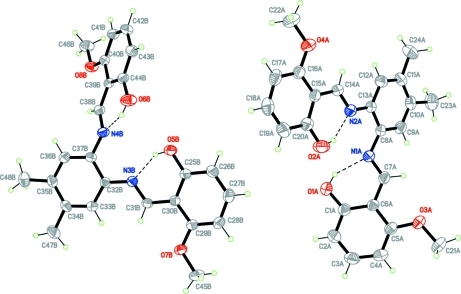
The asymmetric unit of the title compound, showing 40% probability displacement ellipsoids and the atomic numbering. Intramolecular hydrogen bonds are drawn as dashed lines.

**Fig. 2.**
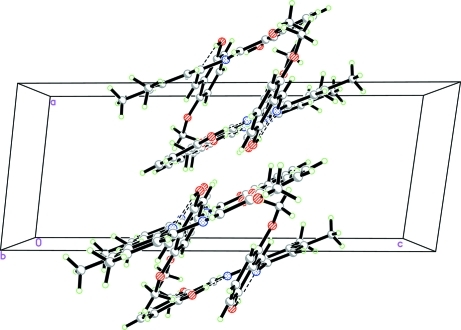
A part of the crystal packing of the title compound viewed down the b-axis.

## supplementary crystallographic information

### Comment

Schiff base ligands are one of the most prevalent systems in coordination chemistry. As part of a general study of tetradenate Schiff bases (Kargar *et al.,* 2009; Kargar *et al.* 2010**a*,b*; Kia *et al.* 2010; Kia *et al.,* 2011), we have determined the crystal structure of the title compound.

The asymmetric unit of the title compound, Fig. 1, comprises two crystallographically independent molecules A and B. The bond lengths (Allen *et al.,* 1987) and angles are within the normal ranges The dihedral angles between the central dimethyl-substituted phenyl ring with the two outer phenyl rings are 49.5 (1) and 5.06 (11)° in molecule A and 5.77 (9) and 42.55 (8)° in molecule B, respectively. Four strong intramolecular O—H···N hydrogen bonds (Table 1) generate four *S(6)* ring motifs (Bernstein *et al.,* 1995). The crystal structure is further stabilized by the intermolecular π-π interactions [*Cg*1··· *Cg*1^i^ = 3.7608 (13)Å, (i) 1 - x, 1 - y, 2 - z; *Cg*2··· *Cg*3^ii^ = 3.8765 (12)Å, (ii) 1 - x, -y, 2 - z; *Cg*4··· *Cg*4^iii^ = 3.5913 (10)Å, (ii) -x, -y, 1 - z, *Cg*1, *Cg*2, *Cg*3, and *Cg*4 are the centroids of  C(1A)–C(6A), C(8A)–C(13A), C(15A)–C(20A), and  C(39B)–C(44B) rings.

### Experimental

The title compound was synthesized by adding 6-methoxy-salicylaldehyde (4 mmol) to a solution of 4,5-dimethyl-1,2-phenylenediamine (2 mmol) in ethanol (20 ml). The mixture was refluxed with stirring for half an hour. The resultant yellow solution was filtered. Yellow single crystals of the title compound suitable for *X*-ray structure determination were recrystallized from ethanol by slow evaporation of the solvents at room temperature over several days.

### Refinement

H atoms of the hydroxy groups were positioned by a constraied rotating model with U_iso_(H) = 1.5 U_eq_(O), see Table 1. The remaining H atoms were positioned geometrically with C—H = 0.93–0.96Å and included in a riding model approximation with U_iso_ (H) = 1.2 or 1.5 U_eq_ (C). A rotating group model was used for the methyl groups.

### Crystal data

**Table:**

C_24_H_24_N_2_O_4_	*Z* = 4
*M**_r_* = 404.45	*F*(000) = 856
Triclinic, *P*1	*D*_x_ = 1.315 Mg m^−^^3^
Hall symbol: -P 1	Mo *K*α radiation, λ = 0.71073 Å
*a* = 8.0311 (2) Å	Cell parameters from 7158 reflections
*b* = 12.5836 (3) Å	θ = 2.5–24.0°
*c* = 20.6174 (5) Å	µ = 0.09 mm^−^^1^
α = 86.900 (1)°	*T* = 296 K
β = 82.549 (1)°	Block, yellow
γ = 81.806 (1)°	0.32 × 0.15 × 0.11 mm
*V* = 2043.57 (9) Å^3^	

### Data collection

**Table:**

Bruker SMART APEXII CCD area-detector diffractometer	10103 independent reflections
Radiation source: fine-focus sealed tube	5848 reflections with *I* > 2˘*I*)
graphite	*R*_int_ = 0.044
φ and ω scans	θ_max_ = 28.4°, θ_min_ = 1.6°
Absorption correction: multi-scan (*SADABS*; Bruker, 2005)	*h* = −7→10
*T*_min_ = 0.972, *T*_max_ = 0.990	*k* = −16→16
36786 measured reflections	*l* = −27→27

### Refinement

**Table:**

Refinement on *F*^2^	Primary atom site location: structure-invariant direct methods
Least-squares matrix: full	Secondary atom site location: difference Fourier map
*R*[*F*^2^ > 2σ(*F*^2^)] = 0.053	Hydrogen site location: inferred from neighbouring sites
*wR*(*F*^2^) = 0.149	H-atom parameters constrained
*S* = 1.01	*w* = 1/[σ^2^(*F*_o_^2^) + (0.0638*P*)^2^ + 0.3683*P*] where *P* = (*F*_o_^2^ + 2*F*_c_^2^)/3
10103 reflections	(Δ/σ)_max_ = 0.001
553 parameters	Δρ_max_ = 0.30 e Å^−^^3^
0 restraints	Δρ_min_ = −0.20 e Å^−^^3^

### Special details

**Table:**

Geometry. All esds (except the esd in the dihedral angle between two l.s. planes) are estimated using the full covariance matrix. The cell esds are taken into account individually in the estimation of esds in distances, angles and torsion angles; correlations between esds in cell parameters are only used when they are defined by crystal symmetry. An approximate (isotropic) treatment of cell esds is used for estimating esds involving l.s. planes.
Refinement. Refinement of F^2^ against ALL reflections. The weighted R-factor wR and goodness of fit S are based on F^2^, conventional R-factors R are based on F, with F set to zero for negative F^2^. The threshold expression of F^2^ > 2sigma(F^2^) is used only for calculating R-factors(gt) etc. and is not relevant to the choice of reflections for refinement. R-factors based on F^2^ are statistically about twice as large as those based on F, and R- factors based on ALL data will be even larger.

### Fractional atomic coordinates and isotropic or equivalent isotropic displacement parameters (Å^2^)

**Table:**

	*x*	*y*	*z*	*U*_iso_*/*U*_eq_	
O1A	0.1615 (2)	0.40459 (12)	0.92569 (7)	0.0655 (4)	
H1	0.1569	0.3464	0.9453	0.098*	
O2A	0.4672 (2)	0.20064 (12)	0.87533 (8)	0.0766 (5)	
H2	0.4102	0.1825	0.9090	0.115*	
O3A	0.3431 (2)	0.48135 (11)	1.12519 (7)	0.0643 (4)	
O4A	0.4790 (2)	−0.16724 (11)	0.83813 (7)	0.0676 (4)	
O5B	0.3083 (2)	0.27459 (10)	0.54537 (7)	0.0582 (4)	
H5	0.2627	0.3012	0.5138	0.087*	
O6B	0.36319 (17)	0.09296 (10)	0.44648 (8)	0.0538 (4)	
H6	0.3277	0.1533	0.4330	0.081*	
O7B	0.33691 (17)	0.64232 (9)	0.57086 (6)	0.0472 (3)	
O8B	−0.15187 (15)	0.02857 (9)	0.37853 (6)	0.0441 (3)	
N1A	0.1970 (2)	0.26121 (12)	1.02084 (7)	0.0459 (4)	
N2A	0.3208 (2)	0.07623 (12)	0.95799 (7)	0.0480 (4)	
N3B	0.20177 (18)	0.41821 (11)	0.46432 (7)	0.0368 (3)	
N4B	0.15172 (18)	0.24553 (11)	0.39805 (7)	0.0374 (3)	
C1A	0.2120 (3)	0.47308 (15)	0.96424 (10)	0.0488 (5)	
C2A	0.2250 (3)	0.57696 (17)	0.94043 (11)	0.0616 (6)	
H2A	0.1960	0.5983	0.8990	0.074*	
C3A	0.2801 (3)	0.64792 (17)	0.97760 (12)	0.0663 (6)	
H3A	0.2897	0.7171	0.9606	0.080*	
C4A	0.3220 (3)	0.62050 (16)	1.03942 (11)	0.0593 (6)	
H4A	0.3601	0.6700	1.0639	0.071*	
C5A	0.3064 (2)	0.51812 (16)	1.06433 (10)	0.0480 (5)	
C6A	0.2518 (2)	0.44164 (15)	1.02743 (9)	0.0434 (4)	
C7A	0.2365 (2)	0.33490 (15)	1.05401 (9)	0.0452 (5)	
H7A	0.2562	0.3188	1.0971	0.054*	
C8A	0.1648 (2)	0.16144 (15)	1.05199 (9)	0.0421 (4)	
C9A	0.0701 (3)	0.15609 (17)	1.11315 (9)	0.0509 (5)	
H9A	0.0333	0.2193	1.1353	0.061*	
C10A	0.0286 (3)	0.06034 (18)	1.14232 (10)	0.0518 (5)	
C11A	0.0802 (3)	−0.03431 (16)	1.10848 (10)	0.0518 (5)	
C12A	0.1752 (3)	−0.02903 (16)	1.04781 (10)	0.0525 (5)	
H12A	0.2106	−0.0921	1.0254	0.063*	
C13A	0.2203 (2)	0.06715 (15)	1.01883 (9)	0.0427 (4)	
C14A	0.3726 (2)	−0.00173 (15)	0.91988 (9)	0.0456 (5)	
H14A	0.3472	−0.0701	0.9328	0.055*	
C15A	0.4693 (2)	0.01451 (15)	0.85743 (9)	0.0439 (4)	
C16A	0.5221 (3)	−0.07042 (16)	0.81420 (9)	0.0490 (5)	
C17A	0.6084 (3)	−0.05346 (19)	0.75346 (10)	0.0594 (6)	
H17A	0.6423	−0.1100	0.7253	0.071*	
C18A	0.6442 (3)	0.0486 (2)	0.73471 (10)	0.0639 (6)	
H18A	0.7017	0.0601	0.6934	0.077*	
C19A	0.5979 (3)	0.13263 (19)	0.77483 (11)	0.0650 (6)	
H19A	0.6245	0.2004	0.7610	0.078*	
C20A	0.5104 (3)	0.11693 (16)	0.83664 (10)	0.0522 (5)	
C21A	0.4026 (4)	0.5531 (2)	1.16515 (12)	0.0794 (8)	
H21A	0.4231	0.5176	1.2063	0.119*	
H21B	0.3188	0.6151	1.1726	0.119*	
H21C	0.5060	0.5751	1.1435	0.119*	
C22A	0.5095 (4)	−0.25444 (19)	0.79537 (13)	0.0937 (9)	
H22A	0.4697	−0.3166	0.8179	0.141*	
H22B	0.6289	−0.2700	0.7815	0.141*	
H22C	0.4505	−0.2357	0.7578	0.141*	
C23A	−0.0699 (3)	0.0594 (2)	1.20956 (11)	0.0736 (7)	
H23A	−0.1038	0.1319	1.2232	0.110*	
H23B	0.0001	0.0208	1.2399	0.110*	
H23C	−0.1687	0.0250	1.2086	0.110*	
C24A	0.0333 (3)	−0.14094 (18)	1.13633 (12)	0.0742 (7)	
H24A	−0.0874	−0.1353	1.1472	0.111*	
H24B	0.0871	−0.1604	1.1750	0.111*	
H24C	0.0705	−0.1949	1.1045	0.111*	
C25B	0.3553 (2)	0.35234 (14)	0.57787 (9)	0.0398 (4)	
C26B	0.4361 (3)	0.32414 (15)	0.63297 (10)	0.0504 (5)	
H26A	0.4591	0.2524	0.6463	0.060*	
C27B	0.4814 (3)	0.40265 (16)	0.66741 (10)	0.0533 (5)	
H27A	0.5354	0.3833	0.7044	0.064*	
C28B	0.4495 (2)	0.51037 (15)	0.64902 (9)	0.0460 (5)	
H28A	0.4800	0.5625	0.6737	0.055*	
C29B	0.3723 (2)	0.53911 (13)	0.59388 (8)	0.0361 (4)	
C30B	0.3236 (2)	0.46071 (13)	0.55645 (8)	0.0327 (4)	
C31B	0.2480 (2)	0.48966 (13)	0.49754 (8)	0.0347 (4)	
H31A	0.2322	0.5613	0.4832	0.042*	
C32B	0.1357 (2)	0.43933 (13)	0.40431 (8)	0.0327 (4)	
C33B	0.0981 (2)	0.54115 (13)	0.37580 (9)	0.0385 (4)	
H33A	0.1166	0.6004	0.3976	0.046*	
C34B	0.0344 (2)	0.55820 (14)	0.31619 (9)	0.0407 (4)	
C35B	0.0057 (2)	0.46919 (15)	0.28374 (9)	0.0428 (4)	
C36B	0.0440 (2)	0.36764 (14)	0.31152 (9)	0.0416 (4)	
H36A	0.0268	0.3085	0.2893	0.050*	
C37B	0.1071 (2)	0.35046 (13)	0.37140 (8)	0.0345 (4)	
C38B	0.0596 (2)	0.17277 (13)	0.39149 (8)	0.0349 (4)	
H38A	−0.0401	0.1925	0.3728	0.042*	
C39B	0.1025 (2)	0.06190 (12)	0.41158 (8)	0.0327 (4)	
C40B	−0.0085 (2)	−0.01310 (13)	0.40432 (8)	0.0340 (4)	
C41B	0.0316 (2)	−0.11995 (14)	0.42275 (9)	0.0421 (4)	
H41A	−0.0420	−0.1692	0.4185	0.051*	
C42B	0.1835 (2)	−0.15232 (14)	0.44774 (9)	0.0449 (5)	
H42A	0.2108	−0.2243	0.4599	0.054*	
C43B	0.2948 (2)	−0.08251 (14)	0.45517 (9)	0.0421 (4)	
H43A	0.3964	−0.1068	0.4717	0.050*	
C44B	0.2538 (2)	0.02470 (13)	0.43775 (8)	0.0368 (4)	
C45B	0.3907 (3)	0.72410 (15)	0.60485 (11)	0.0535 (5)	
H45A	0.3635	0.7924	0.5829	0.080*	
H45B	0.5110	0.7094	0.6058	0.080*	
H45C	0.3340	0.7258	0.6488	0.080*	
C46B	−0.2703 (3)	−0.04164 (16)	0.36976 (11)	0.0553 (5)	
H46A	−0.3665	−0.0014	0.3524	0.083*	
H46B	−0.3067	−0.0751	0.4111	0.083*	
H46C	−0.2177	−0.0959	0.3398	0.083*	
C47B	−0.0004 (3)	0.67051 (16)	0.28798 (11)	0.0607 (6)	
H47A	0.0333	0.7199	0.3160	0.091*	
H47B	−0.1193	0.6881	0.2846	0.091*	
H47C	0.0626	0.6753	0.2453	0.091*	
C48B	−0.0648 (3)	0.48102 (19)	0.21901 (10)	0.0668 (6)	
H48A	−0.0799	0.4116	0.2054	0.100*	
H48B	0.0127	0.5126	0.1868	0.100*	
H48C	−0.1720	0.5264	0.2236	0.100*	

### Atomic displacement parameters (Å^2^)

**Table:**

	*U*^11^	*U*^22^	*U*^33^	*U*^12^	*U*^13^	*U*^23^
O1A	0.0961 (12)	0.0544 (9)	0.0514 (9)	−0.0130 (9)	−0.0264 (8)	−0.0013 (7)
O2A	0.1169 (15)	0.0480 (9)	0.0621 (10)	−0.0258 (9)	0.0182 (9)	−0.0091 (7)
O3A	0.0927 (12)	0.0568 (9)	0.0498 (9)	−0.0199 (8)	−0.0179 (8)	−0.0117 (7)
O4A	0.0996 (12)	0.0452 (8)	0.0569 (9)	−0.0060 (8)	−0.0045 (8)	−0.0139 (7)
O5B	0.0943 (11)	0.0323 (7)	0.0568 (9)	−0.0174 (7)	−0.0350 (8)	0.0052 (6)
O6B	0.0497 (8)	0.0384 (7)	0.0792 (10)	−0.0080 (6)	−0.0283 (7)	−0.0013 (7)
O7B	0.0640 (9)	0.0312 (7)	0.0513 (8)	−0.0108 (6)	−0.0200 (6)	−0.0029 (6)
O8B	0.0419 (7)	0.0368 (7)	0.0583 (8)	−0.0125 (6)	−0.0168 (6)	0.0010 (6)
N1A	0.0574 (10)	0.0401 (9)	0.0409 (9)	−0.0062 (7)	−0.0066 (7)	−0.0058 (7)
N2A	0.0629 (11)	0.0422 (9)	0.0382 (9)	−0.0084 (8)	−0.0013 (8)	−0.0045 (7)
N3B	0.0435 (8)	0.0329 (8)	0.0370 (8)	−0.0093 (6)	−0.0122 (6)	0.0010 (6)
N4B	0.0414 (8)	0.0302 (8)	0.0431 (8)	−0.0068 (6)	−0.0121 (7)	−0.0032 (6)
C1A	0.0529 (12)	0.0432 (11)	0.0506 (12)	−0.0028 (9)	−0.0102 (9)	−0.0057 (9)
C2A	0.0744 (15)	0.0502 (13)	0.0601 (14)	−0.0037 (11)	−0.0160 (11)	0.0041 (10)
C3A	0.0789 (16)	0.0409 (12)	0.0784 (17)	−0.0068 (11)	−0.0107 (13)	0.0035 (11)
C4A	0.0658 (14)	0.0436 (12)	0.0699 (15)	−0.0088 (10)	−0.0073 (11)	−0.0120 (11)
C5A	0.0475 (11)	0.0480 (11)	0.0480 (12)	−0.0033 (9)	−0.0035 (9)	−0.0110 (9)
C6A	0.0447 (11)	0.0401 (10)	0.0445 (11)	−0.0018 (8)	−0.0043 (8)	−0.0074 (8)
C7A	0.0500 (11)	0.0469 (11)	0.0382 (10)	−0.0033 (9)	−0.0057 (8)	−0.0069 (8)
C8A	0.0489 (11)	0.0426 (10)	0.0358 (10)	−0.0060 (9)	−0.0092 (8)	−0.0019 (8)
C9A	0.0561 (12)	0.0537 (12)	0.0420 (11)	−0.0050 (10)	−0.0031 (9)	−0.0081 (9)
C10A	0.0477 (12)	0.0672 (14)	0.0399 (11)	−0.0081 (10)	−0.0050 (9)	0.0029 (10)
C11A	0.0521 (12)	0.0536 (12)	0.0488 (12)	−0.0080 (10)	−0.0091 (9)	0.0131 (10)
C12A	0.0635 (13)	0.0428 (11)	0.0499 (12)	−0.0062 (10)	−0.0045 (10)	−0.0003 (9)
C13A	0.0507 (11)	0.0426 (10)	0.0352 (10)	−0.0068 (9)	−0.0061 (8)	−0.0016 (8)
C14A	0.0584 (12)	0.0378 (10)	0.0413 (11)	−0.0073 (9)	−0.0076 (9)	−0.0030 (8)
C15A	0.0495 (11)	0.0462 (11)	0.0361 (10)	−0.0040 (9)	−0.0079 (8)	−0.0043 (8)
C16A	0.0549 (12)	0.0478 (12)	0.0441 (11)	0.0010 (9)	−0.0121 (9)	−0.0065 (9)
C17A	0.0634 (14)	0.0699 (15)	0.0415 (12)	0.0064 (11)	−0.0061 (10)	−0.0152 (10)
C18A	0.0659 (15)	0.0836 (17)	0.0381 (11)	−0.0038 (13)	0.0001 (10)	0.0010 (11)
C19A	0.0784 (16)	0.0643 (14)	0.0503 (13)	−0.0159 (12)	0.0017 (11)	0.0072 (11)
C20A	0.0632 (13)	0.0493 (12)	0.0441 (11)	−0.0094 (10)	−0.0033 (10)	−0.0043 (9)
C21A	0.108 (2)	0.0736 (17)	0.0654 (16)	−0.0224 (15)	−0.0234 (14)	−0.0259 (13)
C22A	0.146 (3)	0.0554 (15)	0.0787 (19)	−0.0001 (16)	−0.0140 (18)	−0.0263 (13)
C23A	0.0689 (16)	0.0964 (19)	0.0526 (14)	−0.0146 (14)	0.0040 (11)	0.0031 (13)
C24A	0.0825 (17)	0.0614 (15)	0.0742 (16)	−0.0118 (13)	−0.0012 (13)	0.0222 (12)
C25B	0.0483 (11)	0.0331 (9)	0.0408 (10)	−0.0099 (8)	−0.0112 (8)	0.0003 (7)
C26B	0.0649 (13)	0.0380 (10)	0.0508 (12)	−0.0052 (9)	−0.0217 (10)	0.0060 (9)
C27B	0.0640 (13)	0.0536 (12)	0.0468 (11)	−0.0065 (10)	−0.0266 (10)	0.0017 (9)
C28B	0.0538 (12)	0.0436 (11)	0.0453 (11)	−0.0118 (9)	−0.0164 (9)	−0.0069 (8)
C29B	0.0355 (9)	0.0341 (9)	0.0396 (10)	−0.0075 (7)	−0.0044 (7)	−0.0028 (7)
C30B	0.0331 (9)	0.0323 (9)	0.0337 (9)	−0.0077 (7)	−0.0044 (7)	−0.0013 (7)
C31B	0.0380 (10)	0.0287 (9)	0.0380 (9)	−0.0065 (7)	−0.0059 (7)	0.0010 (7)
C32B	0.0328 (9)	0.0333 (9)	0.0324 (9)	−0.0054 (7)	−0.0058 (7)	0.0006 (7)
C33B	0.0443 (10)	0.0299 (9)	0.0419 (10)	−0.0062 (8)	−0.0058 (8)	−0.0022 (7)
C34B	0.0424 (10)	0.0382 (10)	0.0389 (10)	−0.0023 (8)	−0.0024 (8)	0.0078 (8)
C35B	0.0461 (11)	0.0478 (11)	0.0352 (10)	−0.0075 (9)	−0.0089 (8)	0.0054 (8)
C36B	0.0468 (11)	0.0410 (10)	0.0399 (10)	−0.0106 (8)	−0.0107 (8)	−0.0038 (8)
C37B	0.0342 (9)	0.0307 (9)	0.0395 (10)	−0.0052 (7)	−0.0077 (7)	0.0010 (7)
C38B	0.0356 (9)	0.0329 (9)	0.0368 (9)	−0.0038 (7)	−0.0073 (7)	−0.0028 (7)
C39B	0.0374 (9)	0.0296 (9)	0.0316 (9)	−0.0055 (7)	−0.0036 (7)	−0.0044 (7)
C40B	0.0374 (9)	0.0332 (9)	0.0316 (9)	−0.0051 (7)	−0.0032 (7)	−0.0029 (7)
C41B	0.0483 (11)	0.0342 (10)	0.0454 (11)	−0.0118 (8)	−0.0041 (9)	−0.0027 (8)
C42B	0.0559 (12)	0.0290 (9)	0.0481 (11)	−0.0021 (9)	−0.0056 (9)	0.0023 (8)
C43B	0.0430 (10)	0.0381 (10)	0.0441 (10)	0.0017 (8)	−0.0100 (8)	0.0003 (8)
C44B	0.0403 (10)	0.0332 (9)	0.0387 (10)	−0.0065 (8)	−0.0083 (8)	−0.0046 (7)
C45B	0.0562 (13)	0.0355 (10)	0.0734 (14)	−0.0096 (9)	−0.0175 (11)	−0.0115 (9)
C46B	0.0483 (12)	0.0474 (12)	0.0765 (15)	−0.0180 (10)	−0.0198 (10)	0.0012 (10)
C47B	0.0795 (16)	0.0448 (12)	0.0546 (13)	0.0001 (11)	−0.0120 (11)	0.0138 (10)
C48B	0.0860 (17)	0.0725 (15)	0.0456 (12)	−0.0111 (13)	−0.0266 (12)	0.0077 (11)

### Geometric parameters (Å, °)

**Table:**

O1A—C1A	1.340 (2)	C21A—H21A	0.9600
O1A—H1	0.8200	C21A—H21B	0.9600
O2A—C20A	1.335 (2)	C21A—H21C	0.9600
O2A—H2	0.8200	C22A—H22A	0.9600
O3A—C5A	1.367 (2)	C22A—H22B	0.9600
O3A—C21A	1.424 (2)	C22A—H22C	0.9600
O4A—C16A	1.363 (2)	C23A—H23A	0.9600
O4A—C22A	1.419 (2)	C23A—H23B	0.9600
O5B—C25B	1.338 (2)	C23A—H23C	0.9600
O5B—H5	0.8200	C24A—H24A	0.9600
O6B—C44B	1.347 (2)	C24A—H24B	0.9600
O6B—H6	0.8200	C24A—H24C	0.9600
O7B—C29B	1.362 (2)	C25B—C26B	1.385 (3)
O7B—C45B	1.421 (2)	C25B—C30B	1.409 (2)
O8B—C40B	1.356 (2)	C26B—C27B	1.363 (3)
O8B—C46B	1.421 (2)	C26B—H26A	0.9300
N1A—C7A	1.279 (2)	C27B—C28B	1.385 (3)
N1A—C8A	1.420 (2)	C27B—H27A	0.9300
N2A—C14A	1.279 (2)	C28B—C29B	1.372 (2)
N2A—C13A	1.409 (2)	C28B—H28A	0.9300
N3B—C31B	1.282 (2)	C29B—C30B	1.411 (2)
N3B—C32B	1.406 (2)	C30B—C31B	1.434 (2)
N4B—C38B	1.279 (2)	C31B—H31A	0.9300
N4B—C37B	1.418 (2)	C32B—C33B	1.390 (2)
C1A—C2A	1.384 (3)	C32B—C37B	1.400 (2)
C1A—C6A	1.404 (3)	C33B—C34B	1.386 (2)
C2A—C3A	1.362 (3)	C33B—H33A	0.9300
C2A—H2A	0.9300	C34B—C35B	1.396 (2)
C3A—C4A	1.374 (3)	C34B—C47B	1.501 (2)
C3A—H3A	0.9300	C35B—C36B	1.382 (2)
C4A—C5A	1.377 (3)	C35B—C48B	1.508 (3)
C4A—H4A	0.9300	C36B—C37B	1.389 (2)
C5A—C6A	1.408 (2)	C36B—H36A	0.9300
C6A—C7A	1.439 (3)	C38B—C39B	1.440 (2)
C7A—H7A	0.9300	C38B—H38A	0.9300
C8A—C9A	1.389 (3)	C39B—C44B	1.403 (2)
C8A—C13A	1.390 (2)	C39B—C40B	1.414 (2)
C9A—C10A	1.382 (3)	C40B—C41B	1.382 (2)
C9A—H9A	0.9300	C41B—C42B	1.385 (3)
C10A—C11A	1.397 (3)	C41B—H41A	0.9300
C10A—C23A	1.505 (3)	C42B—C43B	1.367 (3)
C11A—C12A	1.382 (3)	C42B—H42A	0.9300
C11A—C24A	1.509 (3)	C43B—C44B	1.382 (2)
C12A—C13A	1.394 (3)	C43B—H43A	0.9300
C12A—H12A	0.9300	C45B—H45A	0.9600
C14A—C15A	1.435 (3)	C45B—H45B	0.9600
C14A—H14A	0.9300	C45B—H45C	0.9600
C15A—C20A	1.407 (3)	C46B—H46A	0.9600
C15A—C16A	1.411 (3)	C46B—H46B	0.9600
C16A—C17A	1.372 (3)	C46B—H46C	0.9600
C17A—C18A	1.379 (3)	C47B—H47A	0.9600
C17A—H17A	0.9300	C47B—H47B	0.9600
C18A—C19A	1.358 (3)	C47B—H47C	0.9600
C18A—H18A	0.9300	C48B—H48A	0.9600
C19A—C20A	1.392 (3)	C48B—H48B	0.9600
C19A—H19A	0.9300	C48B—H48C	0.9600
			
C1A—O1A—H1	109.5	C11A—C24A—H24A	109.5
C20A—O2A—H2	109.5	C11A—C24A—H24B	109.5
C5A—O3A—C21A	117.95 (17)	H24A—C24A—H24B	109.5
C16A—O4A—C22A	118.15 (18)	C11A—C24A—H24C	109.5
C25B—O5B—H5	109.5	H24A—C24A—H24C	109.5
C44B—O6B—H6	109.5	H24B—C24A—H24C	109.5
C29B—O7B—C45B	117.36 (14)	O5B—C25B—C26B	118.66 (16)
C40B—O8B—C46B	118.32 (14)	O5B—C25B—C30B	120.71 (15)
C7A—N1A—C8A	119.99 (16)	C26B—C25B—C30B	120.62 (16)
C14A—N2A—C13A	124.29 (16)	C27B—C26B—C25B	119.35 (17)
C31B—N3B—C32B	124.33 (14)	C27B—C26B—H26A	120.3
C38B—N4B—C37B	119.21 (14)	C25B—C26B—H26A	120.3
O1A—C1A—C2A	118.50 (18)	C26B—C27B—C28B	122.03 (17)
O1A—C1A—C6A	121.57 (18)	C26B—C27B—H27A	119.0
C2A—C1A—C6A	119.93 (18)	C28B—C27B—H27A	119.0
C3A—C2A—C1A	120.1 (2)	C29B—C28B—C27B	119.13 (17)
C3A—C2A—H2A	120.0	C29B—C28B—H28A	120.4
C1A—C2A—H2A	120.0	C27B—C28B—H28A	120.4
C2A—C3A—C4A	122.0 (2)	O7B—C29B—C28B	124.06 (15)
C2A—C3A—H3A	119.0	O7B—C29B—C30B	115.01 (14)
C4A—C3A—H3A	119.0	C28B—C29B—C30B	120.93 (16)
C3A—C4A—C5A	118.6 (2)	C25B—C30B—C29B	117.91 (15)
C3A—C4A—H4A	120.7	C25B—C30B—C31B	120.75 (14)
C5A—C4A—H4A	120.7	C29B—C30B—C31B	121.33 (15)
O3A—C5A—C4A	124.14 (18)	N3B—C31B—C30B	120.99 (15)
O3A—C5A—C6A	114.44 (17)	N3B—C31B—H31A	119.5
C4A—C5A—C6A	121.42 (19)	C30B—C31B—H31A	119.5
C1A—C6A—C5A	117.95 (18)	C33B—C32B—C37B	118.43 (15)
C1A—C6A—C7A	121.23 (17)	C33B—C32B—N3B	124.74 (15)
C5A—C6A—C7A	120.82 (17)	C37B—C32B—N3B	116.83 (14)
N1A—C7A—C6A	122.80 (17)	C34B—C33B—C32B	122.80 (16)
N1A—C7A—H7A	118.6	C34B—C33B—H33A	118.6
C6A—C7A—H7A	118.6	C32B—C33B—H33A	118.6
C9A—C8A—C13A	118.96 (17)	C33B—C34B—C35B	118.45 (16)
C9A—C8A—N1A	121.67 (17)	C33B—C34B—C47B	119.72 (17)
C13A—C8A—N1A	119.25 (16)	C35B—C34B—C47B	121.83 (17)
C10A—C9A—C8A	122.42 (18)	C36B—C35B—C34B	119.09 (16)
C10A—C9A—H9A	118.8	C36B—C35B—C48B	119.22 (17)
C8A—C9A—H9A	118.8	C34B—C35B—C48B	121.70 (17)
C9A—C10A—C11A	118.86 (18)	C35B—C36B—C37B	122.52 (16)
C9A—C10A—C23A	119.9 (2)	C35B—C36B—H36A	118.7
C11A—C10A—C23A	121.3 (2)	C37B—C36B—H36A	118.7
C12A—C11A—C10A	118.68 (18)	C36B—C37B—C32B	118.70 (15)
C12A—C11A—C24A	119.7 (2)	C36B—C37B—N4B	121.79 (15)
C10A—C11A—C24A	121.60 (19)	C32B—C37B—N4B	119.42 (14)
C11A—C12A—C13A	122.57 (19)	N4B—C38B—C39B	123.34 (16)
C11A—C12A—H12A	118.7	N4B—C38B—H38A	118.3
C13A—C12A—H12A	118.7	C39B—C38B—H38A	118.3
C8A—C13A—C12A	118.48 (17)	C44B—C39B—C40B	118.17 (15)
C8A—C13A—N2A	116.84 (16)	C44B—C39B—C38B	121.95 (15)
C12A—C13A—N2A	124.67 (17)	C40B—C39B—C38B	119.87 (15)
N2A—C14A—C15A	121.13 (18)	O8B—C40B—C41B	124.56 (15)
N2A—C14A—H14A	119.4	O8B—C40B—C39B	114.87 (14)
C15A—C14A—H14A	119.4	C41B—C40B—C39B	120.57 (16)
C20A—C15A—C16A	117.82 (18)	C40B—C41B—C42B	118.74 (16)
C20A—C15A—C14A	120.72 (17)	C40B—C41B—H41A	120.6
C16A—C15A—C14A	121.43 (18)	C42B—C41B—H41A	120.6
O4A—C16A—C17A	124.52 (18)	C43B—C42B—C41B	122.51 (16)
O4A—C16A—C15A	114.31 (17)	C43B—C42B—H42A	118.7
C17A—C16A—C15A	121.2 (2)	C41B—C42B—H42A	118.7
C16A—C17A—C18A	119.1 (2)	C42B—C43B—C44B	118.90 (17)
C16A—C17A—H17A	120.5	C42B—C43B—H43A	120.6
C18A—C17A—H17A	120.5	C44B—C43B—H43A	120.6
C19A—C18A—C17A	122.0 (2)	O6B—C44B—C43B	118.27 (16)
C19A—C18A—H18A	119.0	O6B—C44B—C39B	120.63 (15)
C17A—C18A—H18A	119.0	C43B—C44B—C39B	121.10 (16)
C18A—C19A—C20A	119.8 (2)	O7B—C45B—H45A	109.5
C18A—C19A—H19A	120.1	O7B—C45B—H45B	109.5
C20A—C19A—H19A	120.1	H45A—C45B—H45B	109.5
O2A—C20A—C19A	118.66 (19)	O7B—C45B—H45C	109.5
O2A—C20A—C15A	121.15 (18)	H45A—C45B—H45C	109.5
C19A—C20A—C15A	120.19 (19)	H45B—C45B—H45C	109.5
O3A—C21A—H21A	109.5	O8B—C46B—H46A	109.5
O3A—C21A—H21B	109.5	O8B—C46B—H46B	109.5
H21A—C21A—H21B	109.5	H46A—C46B—H46B	109.5
O3A—C21A—H21C	109.5	O8B—C46B—H46C	109.5
H21A—C21A—H21C	109.5	H46A—C46B—H46C	109.5
H21B—C21A—H21C	109.5	H46B—C46B—H46C	109.5
O4A—C22A—H22A	109.5	C34B—C47B—H47A	109.5
O4A—C22A—H22B	109.5	C34B—C47B—H47B	109.5
H22A—C22A—H22B	109.5	H47A—C47B—H47B	109.5
O4A—C22A—H22C	109.5	C34B—C47B—H47C	109.5
H22A—C22A—H22C	109.5	H47A—C47B—H47C	109.5
H22B—C22A—H22C	109.5	H47B—C47B—H47C	109.5
C10A—C23A—H23A	109.5	C35B—C48B—H48A	109.5
C10A—C23A—H23B	109.5	C35B—C48B—H48B	109.5
H23A—C23A—H23B	109.5	H48A—C48B—H48B	109.5
C10A—C23A—H23C	109.5	C35B—C48B—H48C	109.5
H23A—C23A—H23C	109.5	H48A—C48B—H48C	109.5
H23B—C23A—H23C	109.5	H48B—C48B—H48C	109.5
			
O1A—C1A—C2A—C3A	−178.3 (2)	O5B—C25B—C26B—C27B	−178.60 (19)
C6A—C1A—C2A—C3A	1.6 (3)	C30B—C25B—C26B—C27B	1.6 (3)
C1A—C2A—C3A—C4A	−1.0 (4)	C25B—C26B—C27B—C28B	−0.1 (3)
C2A—C3A—C4A—C5A	−0.4 (4)	C26B—C27B—C28B—C29B	−1.0 (3)
C21A—O3A—C5A—C4A	−0.6 (3)	C45B—O7B—C29B—C28B	2.9 (3)
C21A—O3A—C5A—C6A	178.73 (19)	C45B—O7B—C29B—C30B	−176.97 (15)
C3A—C4A—C5A—O3A	−179.6 (2)	C27B—C28B—C29B—O7B	−179.11 (18)
C3A—C4A—C5A—C6A	1.1 (3)	C27B—C28B—C29B—C30B	0.7 (3)
O1A—C1A—C6A—C5A	179.01 (18)	O5B—C25B—C30B—C29B	178.35 (16)
C2A—C1A—C6A—C5A	−0.9 (3)	C26B—C25B—C30B—C29B	−1.8 (3)
O1A—C1A—C6A—C7A	−1.3 (3)	O5B—C25B—C30B—C31B	−3.1 (3)
C2A—C1A—C6A—C7A	178.80 (19)	C26B—C25B—C30B—C31B	176.74 (18)
O3A—C5A—C6A—C1A	−179.84 (17)	O7B—C29B—C30B—C25B	−179.49 (15)
C4A—C5A—C6A—C1A	−0.5 (3)	C28B—C29B—C30B—C25B	0.6 (3)
O3A—C5A—C6A—C7A	0.5 (3)	O7B—C29B—C30B—C31B	2.0 (2)
C4A—C5A—C6A—C7A	179.84 (18)	C28B—C29B—C30B—C31B	−177.89 (17)
C8A—N1A—C7A—C6A	−172.74 (17)	C32B—N3B—C31B—C30B	−176.26 (15)
C1A—C6A—C7A—N1A	4.6 (3)	C25B—C30B—C31B—N3B	2.6 (3)
C5A—C6A—C7A—N1A	−175.78 (18)	C29B—C30B—C31B—N3B	−178.94 (16)
C7A—N1A—C8A—C9A	42.5 (3)	C31B—N3B—C32B—C33B	−5.8 (3)
C7A—N1A—C8A—C13A	−141.64 (18)	C31B—N3B—C32B—C37B	173.94 (16)
C13A—C8A—C9A—C10A	−0.1 (3)	C37B—C32B—C33B—C34B	−0.2 (3)
N1A—C8A—C9A—C10A	175.80 (18)	N3B—C32B—C33B—C34B	179.44 (17)
C8A—C9A—C10A—C11A	−1.5 (3)	C32B—C33B—C34B—C35B	0.4 (3)
C8A—C9A—C10A—C23A	178.14 (19)	C32B—C33B—C34B—C47B	−179.00 (18)
C9A—C10A—C11A—C12A	1.8 (3)	C33B—C34B—C35B—C36B	−0.9 (3)
C23A—C10A—C11A—C12A	−177.8 (2)	C47B—C34B—C35B—C36B	178.50 (18)
C9A—C10A—C11A—C24A	−177.59 (19)	C33B—C34B—C35B—C48B	179.47 (19)
C23A—C10A—C11A—C24A	2.7 (3)	C47B—C34B—C35B—C48B	−1.1 (3)
C10A—C11A—C12A—C13A	−0.6 (3)	C34B—C35B—C36B—C37B	1.3 (3)
C24A—C11A—C12A—C13A	178.85 (19)	C48B—C35B—C36B—C37B	−179.09 (19)
C9A—C8A—C13A—C12A	1.4 (3)	C35B—C36B—C37B—C32B	−1.1 (3)
N1A—C8A—C13A—C12A	−174.64 (17)	C35B—C36B—C37B—N4B	−177.56 (17)
C9A—C8A—C13A—N2A	−177.88 (17)	C33B—C32B—C37B—C36B	0.6 (2)
N1A—C8A—C13A—N2A	6.1 (3)	N3B—C32B—C37B—C36B	−179.15 (16)
C11A—C12A—C13A—C8A	−1.0 (3)	C33B—C32B—C37B—N4B	177.10 (15)
C11A—C12A—C13A—N2A	178.15 (19)	N3B—C32B—C37B—N4B	−2.6 (2)
C14A—N2A—C13A—C8A	−175.51 (18)	C38B—N4B—C37B—C36B	−38.6 (2)
C14A—N2A—C13A—C12A	5.3 (3)	C38B—N4B—C37B—C32B	145.00 (16)
C13A—N2A—C14A—C15A	177.59 (17)	C37B—N4B—C38B—C39B	174.28 (15)
N2A—C14A—C15A—C20A	0.3 (3)	N4B—C38B—C39B—C44B	−2.6 (3)
N2A—C14A—C15A—C16A	−177.86 (18)	N4B—C38B—C39B—C40B	178.22 (16)
C22A—O4A—C16A—C17A	−6.7 (3)	C46B—O8B—C40B—C41B	0.2 (2)
C22A—O4A—C16A—C15A	172.9 (2)	C46B—O8B—C40B—C39B	−179.90 (16)
C20A—C15A—C16A—O4A	179.36 (17)	C44B—C39B—C40B—O8B	−179.79 (14)
C14A—C15A—C16A—O4A	−2.4 (3)	C38B—C39B—C40B—O8B	−0.6 (2)
C20A—C15A—C16A—C17A	−1.0 (3)	C44B—C39B—C40B—C41B	0.1 (2)
C14A—C15A—C16A—C17A	177.26 (18)	C38B—C39B—C40B—C41B	179.35 (15)
O4A—C16A—C17A—C18A	179.79 (19)	O8B—C40B—C41B—C42B	179.19 (16)
C15A—C16A—C17A—C18A	0.2 (3)	C39B—C40B—C41B—C42B	−0.7 (3)
C16A—C17A—C18A—C19A	0.6 (3)	C40B—C41B—C42B—C43B	0.3 (3)
C17A—C18A—C19A—C20A	−0.5 (4)	C41B—C42B—C43B—C44B	0.7 (3)
C18A—C19A—C20A—O2A	179.6 (2)	C42B—C43B—C44B—O6B	178.77 (17)
C18A—C19A—C20A—C15A	−0.3 (3)	C42B—C43B—C44B—C39B	−1.3 (3)
C16A—C15A—C20A—O2A	−178.89 (19)	C40B—C39B—C44B—O6B	−179.17 (16)
C14A—C15A—C20A—O2A	2.9 (3)	C38B—C39B—C44B—O6B	1.6 (3)
C16A—C15A—C20A—C19A	1.1 (3)	C40B—C39B—C44B—C43B	0.9 (2)
C14A—C15A—C20A—C19A	−177.19 (19)	C38B—C39B—C44B—C43B	−178.31 (16)

### Hydrogen-bond geometry (Å, °)

**Table:**

*D*—H···*A*	*D*—H	H···*A*	*D*···*A*	*D*—H···*A*
O1A—H1···N1A	0.82	1.88	2.608 (2)	147
O2A—H2···N2A	0.82	1.81	2.541 (2)	148
O5B—H5···N3B	0.82	1.79	2.529 (2)	149
O6B—H6···N4B	0.82	1.89	2.621 (2)	148
